# The Influence of Microbiota on Wild Birds’ Parental Coprophagy Behavior: Current Advances and Future Research Directions

**DOI:** 10.3390/microorganisms12122468

**Published:** 2024-11-30

**Authors:** Saba Gul, Yurou Shi, Jie Hu, Sen Song

**Affiliations:** 1 School of Life Sciences, Lanzhou University, Lanzhou 730000, China; 220220949910@lzu.edu.cn (S.G.); shiyr21@lzu.edu.cn (Y.S.); j.hu@cml.leidenuniv.nl (J.H.); 2 Institute of Environmental Sciences, Leiden University, 2333CC Leiden, The Netherlands

**Keywords:** parental coprophagy, microbiota, wild birds, avian biology, conservation

## Abstract

This comprehensive review provides an in-depth exploration of the intriguing phenomenon of parental coprophagy in wild birds and its profound implications on the influence of adult avian parents’ health. This review investigates the composition and dynamics of avian feces’ microbiota, casting light on the various dietary, environmental, and genetic factors that influence its diversity. Furthermore, it emphasizes parental coprophagy, a behavior observed in numerous bird species, particularly among herbivorous and passerine birds. The review investigates multiple hypotheses proposed to explain the occurrence of coprophagy. It delves into its function as a potential mechanism for transmitting microorganisms, particularly feces bacteria, from nestlings to their parents. This microbial transfer may affect the health and well-being of adult avian parents. In addition, the review highlights the current research deficits and debates surrounding coprophagy. These gaps include crucial aspects such as the onset of coprophagy, its long-term effects on both parents and offspring, the nutritional implications of consuming nestling feces, the potential risks of pathogen transmission, and the ecological and evolutionary factors that drive this behavior. As the review synthesizes existing knowledge and identifies areas requiring additional research, it emphasizes the significance of future studies that comprehensively address these gaps. By doing so, we can understand coprophagy’s ecological and evolutionary significance in wild birds, advancing our knowledge on avian biology. This information can improve conservation efforts to protect migratory bird populations and their complex ecosystems.

## 1. Introduction

The microbiota of an organism encompasses a vast array of microorganisms including bacteria, microbial eukaryotes, archaea, and viruses that colonize surfaces such as the skin, or internal sites like the gastrointestinal and respiratory tracts [1]. The collective genomes are commonly called the microbiome [2]. Multiple studies have demonstrated that the health and behavior of hosts are contingent upon a harmonious composition of the microbiome [3,4,5]. Hosts may gain numerous benefits from these microorganisms, such as improved nutrient absorption and metabolism, intestinal morphological development, detoxification, and immune system activation [6,7,8,9]. The health state of an animal host can be reflected by the richness and composition of the gut microbial community and its functional capacity due to the various microbiota-mediated roles inside vertebrates [10]. Consequently, unraveling the processes that shape the formation of host microbiomes has become a focal point of research in microbial ecology and evolutionary ecology [11,12,13,14].

**Figure 1:**
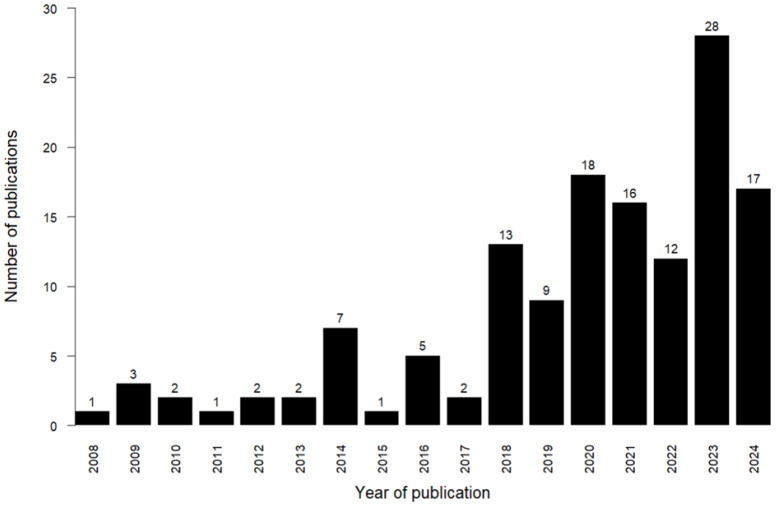
Number of articles published on avian gut microbiota from 2008 to 2024.

Birds play a crucial role in numerous ecosystems, performing a wide range of services such as seed distribution, pollination, pest management (including rodents and insects), and contributing to the availability of soil nutrients [15,16,17]. The microbiota of birds is crucial in maintaining overall health and functioning, similar to other vertebrate hosts [18]. However, our knowledge of the avian microbiota has arguably lagged behind that of many other vertebrates, like humans [2], mice [19,20,21,22], insects, and even fish [23,24]. Based on these observations, it is evident that we need to gain a more comprehensive understanding of the microbial communities associated with birds [25]. In recent years, more research has been conducted on wild avian microbiomes, as seen in Figure 1. This has led to an upsurge in interest in avian gut microbial communities. In Figure 1, we only included studies on wild birds (i.e., excluding captive or domesticated birds) that used 16S rRNA analysis, with literature searches conducted using Google Scholar.

Birds have evolved behavioral strategies to adapt to dynamic environments, increase their offspring’s survival rates, and reduce their losses [26,27]. *Coprophagia* is an animal behavior characterized by the consumption of feces. This includes the consumption of one’s feces (autocoprophagy), the feces of conspecifics (allocoprophagy), or the feces of other species (heterospecific coprophagy) [28]. Coprophagy is commonly observed in canines, pigs, horses, lagomorphs, rodents, and primates, among other mammalian orders [28,29,30]. Allocoprophagy in wild animals typically entails juveniles consuming the feces of adults, which has been hypothesized to facilitate nutrient extraction through food re-digestion and the acquisition of beneficial intestinal symbionts [31,32,33,34]. In birds, coprophagy is most frequently observed in herbivorous precocial species, such as ptarmigans, quails, and turkeys [35,36,37,38], and is a typical behavior also observed in some passerine bird species like Dunnocks (*Prunella modularis*) and Grey-backed shrikes (*Lanius tephronotus*) [39]. Also, in numerous altricial species, parent birds remove the excrement-encased, mucous-coated feces sacs of the nestlings by ingesting them in the nest, a behavior commonly observed in passerines [39,40]. Coprophagia in vertebrates has been opportunistically documented by observing animals [41]. However, studies on the effects of this behavior on the feces’ microbial community are scarce and limited to a few species. Despite the potential significance of coprophagy for the development of diverse animal species, experimental evidence regarding how it affects microbiota, growth, and survival is limited. Studying the effects of parental fecal consumption on microbiota can cast light on the adaptive significance of this behavior, as the microbiota plays an essential role in maintaining the host’s health, including digestion, immune function, and pathogen protection. This paper aims to elucidate the impact of parental ingestion of nestling feces on the gut microbiota of migratory birds, thereby enhancing our comprehension of the microbiota’s contribution to avian health and disease dynamics. We endeavor to present a thorough review of the existing knowledge on how the consumption of nestlings’ fecal matter affects parental gut microbiota in wild avian populations, pinpoint gaps in the current research, and propose avenues for future investigations.

## 2. Bird’s Gut Microbiota

**Figure 2:**
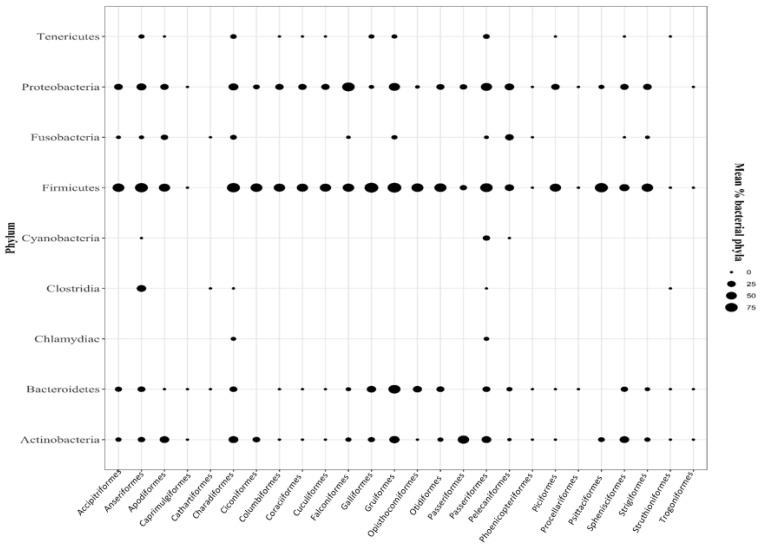
Mean percentages of dominant bacterial phyla in avian orders.

The term ‘bird microbiome’ broadly encompasses a diverse range of microorganisms, mainly bacteria, microbial eukaryotes, archaea, and viruses found in various avian biological niches, such as the gut, skin, or respiratory tract [1]. By taking this comprehensive view, we can delve deeper into the existing literature to uncover studies that provide insights into these complex microbial ecosystems within birds. The DNA sequence data generated by molecular microbiome surveys are classified into operational taxonomic units (OTUs), defined based on a 97% sequence similarity of the 16S rRNA gene, and corresponding to species determined by sequence similarity. There can be thousands of microbial OTUs within the gastrointestinal tract of a single wild avian. Recent advancements in DNA sequencing techniques and concurrent cost reductions have made microbiome research significantly more feasible [4,42,43]. The gut microbiome composition of avian species is typically determined by sequencing the V3-V6 regions of the 16S rRNA gene using DNA extracted from fecal samples [44,45,46,47,48,49]. The bacterial makeup of the bird differs from that of humans, invertebrates, and fish and is more similar to that of reptiles [42]. In addition, the diversity and composition of the avian gastrointestinal microbiota can vary considerably among avian orders as shown in Figure 2. The authors of [50] report that the gut organisms of New Zealand kakapo parrots consist of only a few phyla, whereas those of hoatzins belong to over 40 phyla. To accurately characterize the core microbiota inherent to avian species, it is imperative to analyze and compare the microbial diversity across a broad spectrum of native bird populations.

### 2.1. Microbial Phyla in Bird

#### 2.1.1. Firmicutes

Firmicutes, a phylum of gram-positive bacteria, encompass a diverse array of species. These bacteria are known for fermenting indigestible carbohydrates to produce short-chain fatty acids, which can be readily absorbed through the gut wall and utilized as an energy source by the host [51]. Several Firmicutes pathogens, including *Mycoplasma gallisepticum*, *Clostridium botulinum*, and *C. perfringens*, have been isolated from feral birds [52]. Firmicutes appear to predominate in the gastrointestinal microbiota of birds [8,42,53], an observation that suggests a consistent and specialized microbial community in the bird gut, potentially offering enhanced adaptation to environmental pressures [42].

#### 2.1.2. Proteobacteria

Proteobacteria is a bacterial phylum that contains numerous gram-negative species. They are abundant in a variety of habitats, including the microbiota of the intestine. Birds host a higher proportion of Proteobacteria in comparison to mammals or domestic chickens. This phylum includes several genera that are known to harbor opportunistic pathogens. Notable among these are the Campylobacter, Escherichia, Helicobacter, Rickettsia, Salmonella, and Vibrio, which have all been isolated from avian species [54,55,56,57]. Due to their high taxonomic and functional diversity, the functional significance of Proteobacteria within the avian digestive tract is still unknown. Some Proteobacteria may functionally overlap with other well-studied phyla, whereas others may be transient. Among Proteobacterial classes, α-Proteobacteria are abundant in untamed birds (45%), indicating a need for further research into their function [58].

#### 2.1.3. Actinobacteria

Actinobacteria is a bacterial phylum distinguished by their filamentous structures. They are gram-positive bacteria whose members can inhabit various environments, including soils, fresh and marine waters, and the gastrointestinal tract [59,60]. In addition to pathogens such as Corynebacterium, Mycobacterium, and Nocardia, the Actinobacteria also contain Corynebacterium, Mycobacterium, and Nocardia species. Bifidobacterium is a genus of commensal bacteria [59], and it has been used as a probiotic in humans and animals [61]. The roles of Actinobacteria are critical for sustaining gastrointestinal equilibrium and the broader health of the host organism. Although Actinobacteria constitute the fourth most abundant microbial phylum in the gastrointestinal systems of wild birds, their specific functions within wild and domesticated avian populations remain unexplored in scientific research.

#### 2.1.4. Bacteroidetes

Bacteroidetes is a phylum of gram-negative bacteria prevalent in the intestinal microbiota of animals, including birds. They range from obligate aerobes to strict aerobes. Bacteroidetes populate the entire GI tract of mammals and animals as part of the average GI community [43,62,63]. The authors of [64] report that the three general Bacteroidetes, Prevotella, Porphyromonas, and Flavobacterium, contain potential pathogens of birds and mammals. Bacteroidetes in the avian intestine degrade complex biopolymers, especially polysaccharides such as carbohydrates and plant cell wall components. The comparatively lower abundance of Bacteroidetes in the avian gut compared to that in mammals could be linked to variations in diet, though further investigation is needed to understand this association fully. Nonetheless, studies have shown that Bacteroidetes are more plentiful in the ceca of some bird species, highlighting their potential specialized role in breaking down cellulose and other plant materials. For a definitive examination of this hypothesis, a concurrent analysis of microbial communities across various segments of the gastrointestinal tract in a range of bird species would be essential [64,65,66,67].

## 3. Factors Influencing Microbiota Composition

Birds, the predominant group of winged vertebrates, facilitate connections between geographically remote areas through their migratory and dispersal behaviors [68]. As a result, the composition of their gut microbiota is likely shaped by a complex interplay of internal and external factors [69].

### 3.1. Diet

Dietary composition has been identified as one of the primary determinants of variation in the gut microbiota of mammals [70]. According to [71], the consumption of various forms of food may also be a pathway for colonizing host intestinal microbiota. As in all animals, diet has been found to play an essential role in influencing the avian microbiome [72,73,74,75], but studies of wild birds are scarce, presumably due to the difficulty of assessing diet compositions under uncontrolled conditions. Significant differences in gut microbiota have been found between herbivores, omnivores, and carnivores [58]. Bacteroidetes members frequently dominate microbial communities in the digestive tracts of herbivorous birds. Bacteroidetes can contribute to the breakdown of polysaccharides, cellulose, and other intricate polymers [64]. Therefore, fluctuations in diet can lead to significant alterations in the richness and diversity of the host’s microbiome. A remarkable example is the limited diversity of the intestinal microbiome in vultures, which may be related to their diet carrion [76]. Phylogenetically distant Arctic migratory birds, the Snow Bunting (*Plectrophenax nivalis*) and Sanderling (*Calidris alba*), had more similar microbiome diversity than the herbivorous Pink-footed Geese (*Anser brachyrhynchus*) [77]. In addition, the diversity of intestinal microbial communities was considerably diminished in birds that were fed urban food compared to those that were fed rural food [78]. Dietary differences are believed to play an integral role in shaping the microbial environment; consequently, permanent alterations in diet may induce the colonization of new microbes, thereby increasing the diversity and abundance of beneficial microflora [79].

### 3.2. Environmental Factors

The temporal and spatial heterogeneity of abiotic and biotic variables influences the variation of microbial communities in the environment. Birds are mobile organisms that, depending on the species, require a broad diversity of habitats at various spatial scales [80] and are frequently in mixed-species groups, resulting in overlapping foraging sites and ecological diversity [55,81,82] which significantly affects avian microbial communities, which play a dominant role in molding gastrointestinal microflora, sometimes overshadowing genetic factors. The diversity of microbes within the avian gut has been either directly (e.g., birds acquire microbes from feather preening; [83]) or indirectly (e.g., seasonal or spatial variation in food resources; [84]) related to the landscape or habitat characteristics a species inhabits (e.g., elevation changes; [83]). Human disturbances, like land use, to avian habitats have been found to explain changes in the microbiota of certain avian species [46,49,85,86]. Compared to a breeding area with low levels of human activity, the gut microbiota of swan geese (*Anser cygnoides*) sampled from wintering areas differed in terms of species abundance, interaction network topologies, and pathogen enrichment [87].

### 3.3. Phylogeny and Genetics of Hosts

Genetic composition also significantly impacts the microbial community of the animal intestine, which the progeny can inherit via vertical transmission and co-evolution with the host [88,89]. Consequently, from an evolutionary perspective, similarities in microbial phyla are frequently observed across diverse taxonomic groups (e.g., birds, reptiles, and mammals; [42]). However, avian species exhibit significant variation in the composition and diversity of their microbial communities between species. The distinct life history characteristics and distributions of birds that engage in long-distance migration and the diversity of life-history strategies determine the uniqueness and variation of avian microbiomes [6,8]. In addition, species and evolutionary history distinctions substantially affect the bacterial communities in the animal intestine [58]. Although species-specific gut microbial patterns have been reported in birds, phylosymbiotic signals are weaker in birds compared to non-flying mammals [55,90,91,92,93], which exhibit significant correlations between host phylogenetic divergence and gut microbial characteristics [94]. Phylogeny and diet are likely responsible for differences in the gastrointestinal microbiomes of the closely related Adelie (*Pygoscelis adeliae*) and Gentoo (*P. Papua*) Penguins, which have similar dietary patterns [95].

## 4. Nestling’s Fecal Consumption and How It Links to Gut Microbiota

### 4.1. Parental Coprophagy in Birds

In the context of parental responsibility, parental behavior is defined as any act of parents that enhances the overall health of their progeny and is likely to have evolved and be maintained for this purpose [96]. According to [97], nest sanitation is a crucial but poorly understood aspect of parental care that is prevalent among birds. The removal of nestling excrements, likely one of the essential nest sanitation activities performed by altricial birds [40], has received increased attention in recent years, with a focus on experimental studies investigating the adaptive significance of such behaviors (e.g., [98,99,100]). During the process of nest sanitation, caretakers either remove or ingest the nestlings’ feces. Given that adults are at a greater risk of contracting pathogens when consuming feces [101] it is perplexing why adults consume nestling excrement when they can transport it. To explain the fecal consumption behavior of altricial birds, several hypotheses have been proposed, including the nutrition hypothesis [102], the economic hypothesis [103], and the predation hypothesis [98]. Moreover, a probiotics-related hypothesis was proposed to determine if the Giant babax’s fecal consumption and contest behaviors are related to nestlings’ intestinal microbiota [104].

### 4.2. Coprophagy and the Transfer of Microorganisms

The notion that parental coprophagy in birds may facilitate the transmission of microorganisms from nestlings to adults is an insightful hypothesis. It is suggested that when adult altricial birds consume the feces of their young, they could potentially stabilize their gut microbiota. This proposed mechanism might enhance microbial metabolism and help maintain the energy balance within the host [105]. However, it is important to note that this idea, while intriguing, is currently speculative and would require empirical evidence to be substantiated [104]. Fecal transplantation, either via coprophagy or the oral administration of selected bacteria [106], has also been discussed as a potential method for improving the health of animals in veterinary and wildlife conservation contexts [107,108,109]. For instance, hand-reared Kakapo chicks are occasionally fed frozen feces from adults, but it is unknown whether this practice modifies the juveniles’ microbial communities [110]. There have been a smattering of studies investigating the microbiota of offspring due to the practice of coprophagy, as modern sequencing techniques enable the characterization of microbial communities [105]. Recent research on small mammals suggests that consuming feces may help herbivores maintain the diversity and function of their gastrointestinal microbiome [111]. Ref. [38] studied the development of gut microbiota in wild ptarmigans known to engage in fecal consumption; however, without control groups that were prevented access to maternal feces, the specific function of coprophagia could not be determined. According to [112], coprophagy in ostrich chicks results in accelerated microbiota maturation, increased diversity, decreased pathogen abundance, and enhanced growth and survival, indicating its significance in juvenile development and microbial transmission. Inversely, parental coprophagy carries the danger of transmitting pathogenic microorganisms. As several opportunistic gastrointestinal pathogens that can cause serious disease in birds are spread through the faecal-oral route, including bacteria from the genera Enterococcus, Salmonella, Clostridium, Escherichia, Campylobacter, and Staphylococcus these potentially harmful species may be spread via coprophagy which has been extensively speculated in domestic poultry however, the role of coprophagy in the transmission of pathogens in wild birds and coprophagy has been rarely studied [101]. In conclusion, parental coprophagy in birds is essential for transferring microorganisms, which can have positive and detrimental effects on the health and development of the parents.

### 4.3. Parental Coprophagy and Nutritional Implications

Parental coprophagy, the practice of ingesting their offspring’s feces, can have several nutritional implications for parent birds [113]. The “Parental Nutrition Hypothesis” posits that the digestive systems of juvenile nestlings are initially underdeveloped, leading to many nutrients remaining unabsorbed and thus present in their excrement [113]. With the maturation of juvenile birds, their digestive efficacy advances, enabling them to derive an increased amount of nutrients from their diet [114]. As a result, adult birds discontinue the ingestion of their young’s feces, choosing to remove it from the nest instead [113]. Similar results were observed by [105], in which Grey-backed shrike parents initially consume nestlings’ feces, which contain probiotics and beneficial metabolites, possibly enhancing parental health. However, this behavior ceases as nestlings grow, suggesting a link between fecal content, microbial changes, and parental nutritional benefits. The author of [115] found that fecal sacs are a notable source of protein, calories, and water.

In contrast, the authors of [103] argued that the energy content in the feces indicative of digestive efficacy shows slight variation with fledgling age. This challenges the idea that a reduced energy value is the primary reason parents stop consuming fecal sacs at a young age. However, simply focusing on these arguments may not provide a complete understanding of feces’ nutritional significance to birds. Observations have shown that birds consume feces to obtain essential vitamins [116]. Furthermore, the role of fecal microorganisms in impacting animal nutrition cannot be overlooked, highlighting another potential nutritional avenue for birds [117,118].

## 5. Research Gaps and Controversies

Parental coprophagy in avian behavior remains an area with notable research deficiencies and ongoing debate. Although this practice presents a compelling aspect of bird behavior, many of its intricacies are yet to be fully elucidated. A key area for further investigation is the underlying triggers and mechanisms that prompt parental coprophagy. The precise stimuli or circumstances that elicit this behavior in adult birds remain unclear, calling for more in-depth research for clarification. Moreover, the debate continues as to whether coprophagy is an adaptive behavior specifically triggered by certain stimuli or merely an incidental aspect of nestling care. The dynamics of microbiota during coprophagy represent an additional research lacuna. There is a need for more extensive studies characterizing the microbial communities transferred via parental coprophagy, including the specific microbial species implicated and their functional roles in the development of the nestlings’ microbiome. In addition, there are ongoing debates regarding the extent to which coprophagy influences the long-term composition of the microbiota of nestlings [112].

The nutritional implications of coprophagy require additional research. It is necessary to conduct extensive research to ascertain the precise nutrients transferred via coprophagy and their nutritional impact on parents. Furthermore, discussions persist about the significance of nutrient provisioning via coprophagy and whether this process serves as a critical dietary supplement for the parent birds [105]. The health effects of coprophagy, particularly in terms of pathogen transmission, continue to be a topic of controversy and research interest. Researchers seek to comprehend pathogens’ risks and transmission rates through parental coprophagy, with debates over whether this behavior poses a significant risk or whether the parent’s digestive system neutralizes most of the pathogens [101].

The variation in coprophagy behavior among bird species raises questions about the ecological and evolutionary factors underlying this variation, highlighting the need for comparative research. Methodological challenges further complicate coprophagy research, as collecting data in the wild can be challenging, and the reliability of coprophagy studies is a matter of debate [105].

Addressing these research gaps and controversies will improve our comprehension of parental coprophagy’s ecological, physiological, and evolutionary significance in birds. This will shed light on the intricate relationship between parental health and offspring behavior in avian species.

## 6. Implications and Future Directions

The advancements in modern scientific technologies like Next-Generation Sequencing, Metagenomics, and Stable Isotope Probing (SIP) can help researchers understand the effects of parental fecal consumption on the microbiota of wild bird parents. They offer powerful tools to analyze microbial communities, identify specific microbial taxa, study functional interactions, and assess the potential impacts on bird health and fitness. While research on the effects of parental fecal consumption on the microbiota of wild bird parents is still in its early stages, several potential future directions could be explored to further our understanding of this phenomenon. As our understanding of the microbiota’s role in animal health and fitness grows, there is increasing interest in exploring the effects of parental fecal consumption on the microbiota of wild bird parents. While initial studies have provided intriguing insights into this phenomenon, there is still much to be explored in this field. By exploring the following future directions, researchers can deepen our understanding of the effects of parental fecal consumption on the microbiota of wild bird parents, uncover the underlying mechanisms, and gain insights into this behavior’s ecological and evolutionary implications.

(1) Longitudinal studies: Conducting longitudinal studies that track individual bird parents across multiple reproductive seasons would yield valuable insights into the long-term effects of fecal consumption on the microbiota. This could assist in determining whether the observed alterations in microbiota are temporary or permanent.
(2) Comparative studies: Comparing the gut microbiota of bird species that consume varying amounts of parental feces could cast light on the variation in microbiota dynamics and potential adaptations across species. Investigating closely and distantly associated species with differing levels of parental care could shed light on the evolutionary importance of this behavior.
(3) Health implications: Investigating the ecological variables that govern the incidence and regularity of parental feces consumption among wild bird populations may shed light on the adaptive importance of this behavior. Taking into account factors such as the availability of food resources, breeding density, and prevailing environmental conditions could help achieve an in-depth understanding of the ecological determinants of this practice and its subsequent impact on individual fitness.
(4) Experimental manipulations: Conducting controlled experiments in which the availability or composition of fecal matter is altered could assist in elucidating the causal relationship between fecal consumption and alterations in the microbiota. Providing bird parents with feces from various sources or altering the nutrient content of fecal matter, for instance, could aid in the identification of specific factors that influence microbiota composition.
(5) Concerning evolutionary implications: Understanding the evolutionary significance of parental coprophagy in birds is essential. This behavior is hypothesized to have developed as an adaptive mechanism; however, its role in the broader context of avian evolutionary history is not fully understood. Future research endeavors should focus on the evolutionary consequences of coprophagy, particularly its influence on reproductive outcomes, overall fitness, and species survival. Comparative analyses across different avian taxa could reveal the selective pressures that have contributed to the emergence and maintenance of this behavior over time.

## 7. Conclusions

The study of parental coprophagy in wild birds opens a unique window into avian behavioral ecology, with broad implications for our understanding of their biology and evolution. Adult birds’ ingestion of their nestlings’ feces provides a complex view into the interactions between diet, health, and the environmental pressures that shape these behaviors. While current research indicates that coprophagy may affect avian microbiome balance, nutrition, and disease transmission, much remains to be discovered. Future investigations should adopt longitudinal and cross-species comparative approaches, as well as examine the role of environmental factors in shaping this behavior. Advancing our knowledge of coprophagy will not only deepen our insights into avian ecology but also enhance conservation practices aimed at preserving the delicate balance within bird populations and their ecosystems.
